# Scurrying seafarers: shipboard rats, plague, and the land/sea border

**DOI:** 10.1017/S1740022822000158

**Published:** 2022-05-05

**Authors:** Jules Skotnes-Brown

**Affiliations:** Department of Social Anthropology, University of St Andrews, St Andrews, KY16 9AJ, UK

**Keywords:** rats, ships, oceanic history, maritime history, animal history, medical history

## Abstract

This paper provides a broad overview of spatial, architectural, and sensory relationships between rats and humans on British and American vessels from approximately the 1850s–1950s. Taking rats as my primary historical actors, I show how humans attempted to prevent the movement of these animals between ports across three periods. Firstly, the mid- to- late-nineteenth century, where few attempts were made to prevent rats from boarding ships, and where a multiplicity of human/rat relationships can be located. Secondly, the 1890s–1920s, in which port authorities erected anti-rat borders to lock these animals on land or at sea. Finally, the 1920s–50s, where ships were reconstructed to eliminate all possibilities of rodent inhabitation and to interrupt their transit between ports. Ship rats, I argue, not only demonstrate the fragility of historical rodent-control efforts, but also encourage oceanic historians to consider how animals have negotiated and shaped boundaries between spheres of land and sea.

In 1955, the United States Public Health Service wrote that plague has ‘come to be a maritime disease because it is a disease of rats and rats are great travellers, at least a few of them being on practically every ship’.^1^ Rats in the mid-nineteenth to mid-twentieth centuries were a fundamental part of maritime life. These small rodents gnawed their way through the sinews of global capitalism, inhabited nearly every vessel and terrorised sailors, cats, and dogs. In so doing, rats transgressed boundaries that shaped life on ships, as well as material and conceptual connections between spheres of land and sea. This paper offers a broad overview of the spatial, medical, and sensory relationships between rats and humans in primarily, although not entirely, British and American maritime spaces in the nineteenth and early twentieth centuries. I focus on these maritime powers because of their significance in global naval affairs, and in the management of rats on board ships and in docks. Territorially, economically, and militaristically, British seafaring concerns were of particular importance in this period.^2^ While the United States was also major player in nineteenth and twentieth-century shipping,^3^ what accords it a particularly important role in the story of rats, plague, and maritime capitalism is the presence of its inventors and naval architects.

Within these broader imperial units, this paper does not focus on any specific oceans or groups of sailors, nor does it narrow its source material to any print communities. Instead, it scours travelogues, natural history books, shipping records, and government correspondence for traces of what Kaori Nagai has called ‘vermin writing’ – the ‘ways in which non-human animals emerge as “vermin” in human documents and leave their traces on them’ – on ships and in docks.^4^ In following the maritime traces of rats through numerous archives and across multiple regions, this paper cannot provide a comprehensive minutia of rats on ships. Instead, it offers a panoramic view from which more in-depth histories of rats, infrastructure, and the oceans can be told.

The association of rats and maritime life has a long history. Lucinda Cole has argued that in the early-modern British empire, rats were a ubiquitous part of transatlantic seafaring.^5^ Rats caused great damage to colonies when accidentally imported on ships,^6^ and on board they ravaged food supplies, or attacked sailors directly by nibbling at their toes while they slumbered.^7^ Nagai, likewise, has emphasised the omnipresence of shipboard rats in the nineteenth century.^8^ Yet despite this rodents and other animals in maritime spaces have received little historical attention.^9^ In the last five years, historians have begun to address this lacuna, and a minute but growing literature on shipboard animals has developed. Seafaring animals have been examined as reservoirs of disease,^10^ companion species,^11^ destroyers of the ship and its cargo,^12^ or as objects of commerce.^13^

Studies of nineteenth and twentieth-century seafaring rats stand not only to contribute to maritime history but also to oceanic history in general. In the last two decades, scale and geography have been key concerns of oceanic historians. Scholars have analysed the utility of categories such as the Atlantic or Indian Ocean Worlds, which contain studies within enclosed oceanic units,^14^ and considered the implications of both ‘terracentric’ and ‘aquacentric’ histories.^15^ One of the more interesting developments has been the attempts of scholars to reunite spheres of land and sea.^16^ Far from timeless and natural, these divisions are, as Wilko Graf von Hardenberg argues, ‘recent constructs’;^17^ and products of distinct and complex histories.^18^ In thinking beyond this binary, Alison Bashford’s concept of terraqueous history, which collapses ‘the enduring couplets – land and sea, earth and ocean’, is helpful.^19^ However, when formulating this concept ‘through which to comprehend and consider modern human endeavours where land and water meet’, Bashford neglects the presence of animals.^20^

How non-humans have responded to, engaged with, or even comprehended human-constructed boundaries of land and sea has not been subjected to historical analysis. Seafaring rats, as animals who spend most of their time outside of water yet are adept swimmers who often live on ships, are terraqueous animals of a kind, and an ideal starting point for analysis.^21^ Nestled in bags or barrels of grain, coffee, cotton, and other products, rats in the nineteenth and twentieth centuries hitchhiked across transport networks that connected rural hinterlands with port cities. Finding their way into the holds of ships as stowaways in cargo, or through deliberately climbing mooring ropes and gangways, rats on vessels found themselves in a paradise, replete with food and hiding places. Some rats made ships their permanent homes, while others departed in cargo and established new populations around the world. These globe-scurrying rodents not only demonstrate the frailty of human efforts to control the movement of pests between ports, but by passing between spheres of ship and shore, rats undermined and reshaped physical and conceptual borders between land and sea.^22^

To explore these broader historiographical questions, this paper examines how anti-rodent borders between ships and shores were created, policed, and transformed over approximately the mid-nineteenth to- mid-twentieth centuries. In the process of constructing such boundaries, I argue, humans and rats spatially and architecturally altered the ports that connected disparate lands and seas, as well as the ships that transported goods and people between them. Such changes were ultimately a response to shifting human perceptions of rodent agency. Hence this paper is organised around human experiences of, and attempts to prevent, particular ratty activities in maritime spaces, and how rats circumvented or succumbed to them across three periods:

First, from the mid- to late-nineteenth century, when there was a significant increase in global shipping and mass-migration of both humans and animals.^23^ Here, few attempts were made to curtail the movements of rats from shore to ship, and *gnawing, eating, and drinking* on board ships were identified as their key pestilent maritime activities. From the source material, which is by nature fragmentary, it is impossible to generalise what types of ships these rats were infesting, and even less whether barques, dhows, junks, steamers, and so on were particularly vulnerable to rats. Instead, these pilfering pests were largely accepted as a general feature of maritime life, who upturned rigid spatial hierarchies on board and cohabited with captains, crew, and travellers, alike. While steps could be taken to mitigate the ravages caused by rats’ powerful teeth, little could be done to stop them from crossing to and from spheres of land and sea.

Second, between the 1890s and the 1920s, rodent control shifted towards enforcing a land/sea boundary by preventing rats from *climbing or swimming* on or off board ships. In this period, against the backdrop of the Third Plague Pandemic, rats were rapidly linked to outbreaks of plague, and their presence on board became increasingly unacceptable. With millions of humans and rats dying, various technologies and spatial strategies to remove rats from human maritime spaces were deployed, and international agreements passed to rid ships and docks of ‘vermin’. Lukas Engelman and Christos Lynteris’s *Sulphuric Utopias* provides a compelling analysis of one of these technologies: maritime fumigation. This strategy of distributing poisonous gases into ships to disinfect merchandise and later, to kill rats, was touted as a technology of hygienic modernity, which could create a capitalist utopia of disease-free trade without the need for costly shipping delays caused by maritime quarantine.^24^ However, fumigation was not the only aspect of this desired rat and plague-free utopia: it was paired with infrastructural innovations that attempted to suppress ratty movements between sea and shore. Such innovations focused primarily on preventing black and brown rats from climbing or swimming on board ships.

Third, from the 1920s to the 1950s, the construction of anti-rodent boundaries between land and sea was deemed ineffective and architectural and spatial strategies were developed to prevent not only gnawing, eating, drinking, swimming, and climbing, but also *nesting and movement* on board. In 1920s New York, an ambitious new strategy was attempted: the total rat-proofing of ships. Rat-proofing was rapidly adopted by cargo and passenger shipping companies trading in United States ports and was met with such success that it significantly reduced the need for costly fumigation. While nineteenth century rats moved between ports, disregarding entrenched hierarchies of maritime life, twentieth century rat-proofers attempted to access the spatial and sensory world of rats to identify and remove the spaces that enabled them to thrive, thus making it easy to eliminate on-board survivors. In this period, although enforcing a hard anti-rodent border between ship and shore eventually proved impossible, such technologies succeeded in constructing a new border: one that aimed to prevent rats from travelling between ports.

## Gnawing, Eating, and Drinking: mid- to late- nineteenth-century human/rodent relations before borders

**Figure 1:**
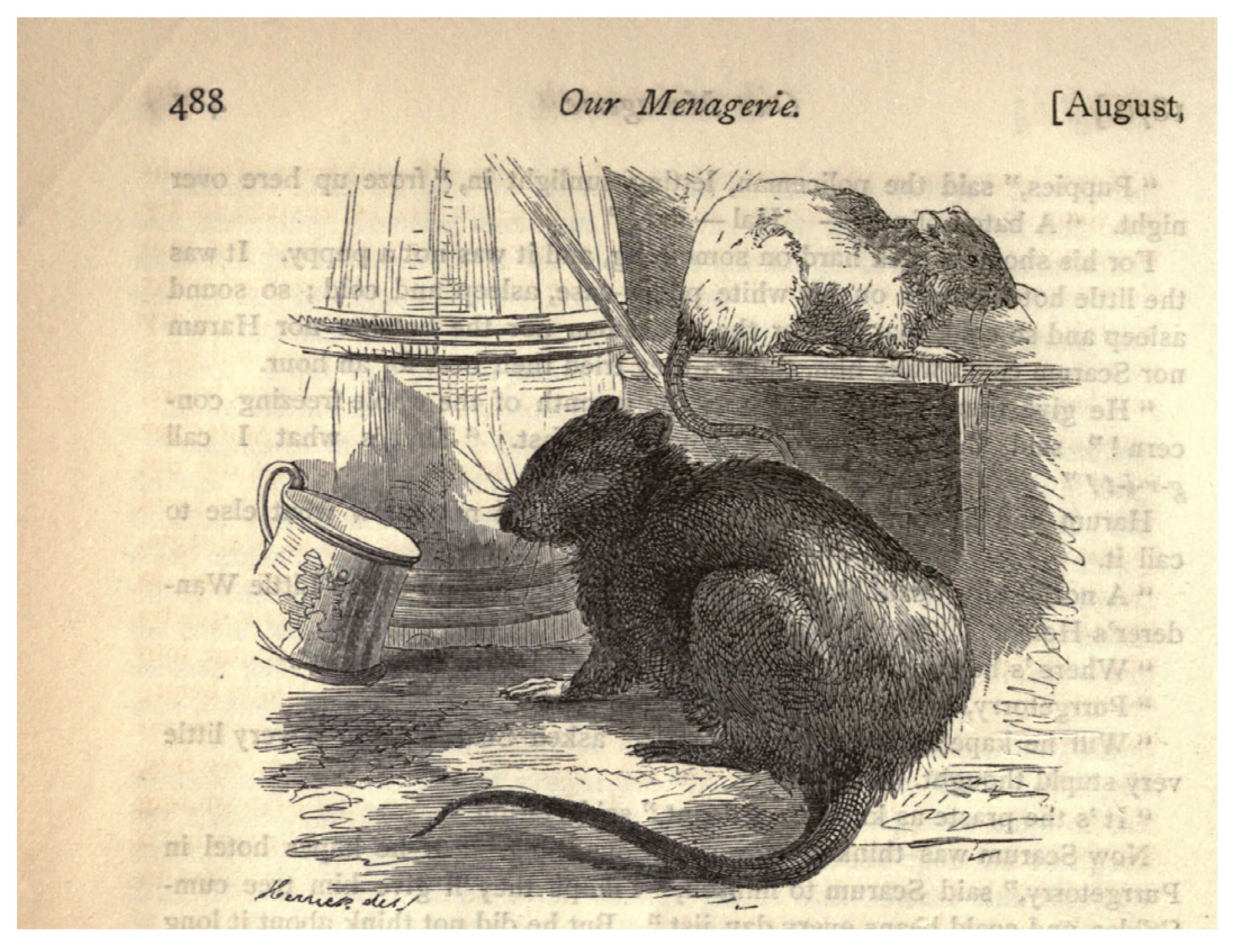
Illustration of two ‘ship rat[s]’ from 1870, August, T.W. Higginson, ‘Our Menagerie. IV.–Rats.’, *Our Young Folks: an Illustrated Magazine for Boys and Girls*, Vol 6, No 8, p 488.

Rats have long been a source of discomfort to sailors and of financial losses to traders. Travelling across supply lines linking cities and rural hinterlands to the international maritime arena, rats frequently found themselves on board vessels where they survived and thrived. Legions of nineteenth-century natural historians, seafarers, and writers in the Anglo world complained bitterly of ship-rats, animals who, according to North American abolitionist, solider, and author Thomas Wentworth Higginson, fared ‘on board ship better than men do’ (Figure 1).^25^ Such writers were largely unconcerned with preventing rats from entering ships, but instead took umbrage at the trespassing of rats into human spaces. Their chief grievances were the definitive activities of rodents: gnawing, eating, and drinking. The very taxonomic category, ‘rodentia’, created by Thomas Edward Bowditch in 1821, was derived from the Latin *rodere*, meaning ‘to gnaw’,^26^ and on ships, black and brown rats put their gnawing prowess to great use. To locate sources of food and water on board, as well as to build nests, rats relied on their sharp incisors, which could perforate almost any wooden shipbuilding or provisions-storage material. Spaces on board in which food, water, and cargo were stored, were particularly vulnerable.^27^ In times of great thirst, according to a court case at the Cape of Good Hope, rats would go as far as to gnaw through ‘*leaden pipes conveying water*’.^28^ In the cargo hold, rats gnawed at anything from bags of coffee to parchment labels affixed to British imperial postage.^29^ At times, rats were indicted for ‘arson’ and ‘larceny’,^30^ or were blamed for chiselling away at the timbers of wooden sailing ships and sprouting leaks.^31^ On occasion, rats even tore sails to pieces, perhaps in search of nesting materials.^32^ On one ship docked in Calcutta in the nineteenth century,^33^ a group of sailors hoisted the sails, and were horrified to witness a ‘perfect shower of rats, old and young … pouring on the deck’.^34^ By gnawing through almost anything, seafaring rats ate away the profits and property of nineteenth century capitalists, and the information networks of the British empire.

Rats inhabiting nineteenth-century vessels not only threatened cargo and architecture but gnawed their way through the rigid spatial hierarchies that structured life on ships. On board eighteenth- to early-twentieth century ships, life was unpleasant for much of the crew and passengers. Conditions were cramped, crowded, nauseating, hot, and often filthy.^35^ Perhaps with the exception of captains and wealthy travellers, there was little privacy for most seafarers, and virtually none at all for labourers or passengers in steerage.^36^ In order to maintain discipline and order in such an unpleasant and potentially incendiary environment, several historians have argued that seventeenth to early twentieth-century ships, whether sail or steam, were structured according to a rigid spatial hierarchy of power, which divided the ship into areas reserved for different social ranks, races, and genders.^37^ Rats utterly disregarded the boundaries between these spaces, moving between places reserved for cargo, the minute sleeping quarters inhabited by Indian seafarers, the packed lower decks of kidnapped Africans on slave ships, or the captain’s cosy chambers, gnawing, eating, drinking, and thriving in the process.

For many passengers on board ships, the human senses were constantly assaulted by disgusting smells, sights, sensations, which rats exacerbated.^38^ The sounds and smells of rats, in particular, were disturbing to many seafarers. In the process of eating, nesting, and scurrying, rats created an unsettling cacophony of noises: scraping, squealing, and scurrying could frequently be heard below the decks and disturbed many a seafarer’s nights rest.^39^ Meanwhile, the smell of a dead and decaying rat, which wafted throughout the ship, was described by one writer as ‘the rankest compound of villainous smells that ever offended nostrils’.^40^ These human sensory experiences provoked by rats sparked emotions that ranged from discomfort to outright horror. Thomas Boteler, a Lieutenant on the HMS Barracouta, while returning to England from a naval survey of the west coast of Africa in 1826, provided a skin-crawling description of sharing his vessel with unwanted pests. Rats,

unable to pick up any refuse of eatables about the deck, attacked the stock of provisions which we had below and made great havoc, besides running over us at night and keeping up a great noise about the decks … Indeed, their ravages [rats and cockroaches] were so unlimited that scarcely anything escaped them. They destroyed our books and clothes, and even attacked us while asleep so ravenously that many of us were latterly obliged to wear gloves, their bites proved very painful, as they sometimes gnawed the skin off for nearly a quarter of an inch square.^41^

These indignities were frequent on British vessels, and captains and wealthy travellers complained bitterly of rats’ invasions into their chambers. Rear-Admiral Beaufort of the HMS Woolwich, for example, was one morning awoken by the disturbing sensation of ‘the cold nose of a rat licking his lips’, who scurried away ‘firmly between the timbers’.^42^ Sinclair Thomas Duncan, a passenger on board a ship returning from Australia via Cape Horn, also experienced a ratty assault. In the 1860s, he wrote that he had been ‘very much annoyed by rats’. As he was drifting into sleep in his cabin, he ‘felt something soft moving along my face’ and upon a scan of the room, spotted a ‘large rat’, and ‘dozens’ of others ‘running about the tables’ which disturbed his rest.^43^ The fact that rats so easily moved between spatial hierarchies on board may have augmented the anxieties provoked by such experiences. Rats were often associated with underclasses, and on ships many commentators thought that they emerged from the lower decks.^44^ By scurrying between the cargo, crew, captain and wealthy travellers’ spaces, ship-rats revealed the close proximities between people across the class and racial spectrum that might otherwise have been hidden on land.

These writings, however, were largely produced by wealthy and literate captains, officers, or travellers, who lived in relative comfort on board ships. Evidence of relationships between rats and labourers or travellers in steerage are more difficult to come by.^45^ Some works of literature suggest that the lower decks were plagued by disturbing sounds made by rats,^46^ while travelogues and periodicals indict rats for making ‘a meal’ of a sailor’s toes,^47^ or gnawing away at ‘the heel of the seaman’.^48^ Indeed, one can imagine that under already cramped conditions, those packed below deck would have experienced ratty assaults of greater frequency and magnitude. Enslaved people, who had no freedom of movement at all, probably fared the worst against ship rats. According to a letter in the *Illustrated London News* recounting the British capture of a slave ship near the coast of Cuba in 1857, the ‘poor captives’ were in a ‘wretched condition’: they were ‘packed closely together, and covered with dirt and vermin’.^49^ Indeed, chained aboard the lower decks on the infamous Middle Passage, and without shoes, enslaved people would have been left relatively defenceless against the night-time gnawing of rats.^50^

Despite the annoyances of rats, attempts to prevent them from boarding ships in the nineteenth century were few. Perhaps this was considered more effort than it was worth, or even impossible.^51^ Instead, rat-control gravitated around killing rats or keeping them away from certain areas on board.^52^ One technique, developed in Egypt, for example, involved submerging a vessel underwater for half an hour to drown the rats on board.^53^ In times of extreme infestation, the services of ratcatchers could also be enlisted.^54^ Cats and dogs were also frequently employed as ratters.^55^ However, their efficacy was often in doubt. In 1857, on board the Dutch ship *Konigin der Nederlanden*, a cat was taken aboard one evening. The morning later, ‘nothing was to be seen but her skin and bones’.^56^ This fileted feline was not the only predator to experience the wrath of starving rats. In the Arctic, American explorer Elisha Kent Kane and his crew were utterly inundated with rats, who became such an aggressive menace that even their dogs ‘were afraid to go into the hold of the vessel.^57^ One dog named Rhina, had her feet and nails ‘ferociously’ gnawed, and Kane was forced to withdraw her from the lower decks, ‘yelping and vanquished’.^58^ At such times, when ratcatchers, dogs, and cats all failed to quell infestations, ships were fumigated with charcoal, sulphur, mercury, or boiling steam as a last resort.^59^

At times, rats were so difficult or expensive to evict from ships, that other seafarers pragmatically attempted to coexist with them. Some attempted to contain them within certain parts of the ship by feeding and watering them in designated spaces.^60^ Other, more superstitious sailors, scorned attempts to kill them, instead describing a rat infestation as a sign of good luck.^61^ At least one captain was not averse to rat companionship, and even brought pet rats on a voyage.^62^ Other seafarers hunted ship rats for sport,^63^ or consumed them as food.^64^ Some even viewed rats as a source of health on board. Before the 1890s, rats were not typically associated with disease.^65^ North American sailor George Barrell, for example, thought that rats performed a ‘service in consuming wastage that might otherwise breed pestilence’ on the decks.^66^ Others, such as Kane, who ate rats as maritime medicine, attributed his ‘comparative immunity from scurvy’ to his regular consumption of rat soup.^67^

Thus, nineteenth century rats gnawed their way into maritime life, feasting upon cargo, disregarding spatial boundaries on board, undermining societal hierarchies when they moved between the spaces of the ship, and inverting the natural order when they predated upon humans, cats and dogs. In so doing, rats disturbed seafarer’s bodies and minds: their noises kept sailors up at night, their rotting corpses reeked, the feeling of a rat running across one’s bed disturbed what little privacy could be had on board and their occasional gnawing away at extremities caused spine-chilling discomfort and pain. To cope with ship rats, humans attempted to restore order by killing (and occasionally eating) them, or by reimagining human and rat relations entirely. In the 1890s, however, this plurality of multispecies relationships on ships would gradually begin to disappear.

## Climbing and Swimming: constructing anti-rodent boundaries

Around the turn of the twentieth century, rats gradually became increasingly unacceptable seafaring companions. This was mainly down to one reason: the global spread of plague. Beginning in Hong Kong in 1894, plague travelled along commercial trade networks to every continent except Antarctica.^68^ By 1959, this pandemic had killed some twelve million people.^69^ As millions of humans and rats died, the tacit acceptance of nineteenth century commentators that it was probably impossible to eliminate rats from maritime trade entirely, was gradually overturned. Here, the problems of rodents devouring cargo, the unpleasant noises, the terrifying sights and sensations they provoked, were quickly subordinated to the lethal dangers they posed to global health.

By the 1890s, ships had long been suspected as reservoirs of infectious diseases, and quarantine restrictions were often imposed on vessels, cargo, and passengers in ports.^70^ In the wake of the rat-flea theory of plague, fears of rats perpetuating the pandemic began to merge with earlier suspicions of ships as unhealthy or diseased spaces.^71^ Accordingly, sailors, public health officials, politicians, shipbuilders, and inventors began to tackle a new challenge: to fundamentally restructure the spatial dynamics of ships and docks to exclude rats from them. To do this, they attempted to construct an anti-rat border between spheres of land and sea by policing two characteristic rat activities: climbing on or off board and swimming to and from shore.

In 1897, the first biomedical study arguing that rats and fleas played a role in the spread of plague was published by Japanese bacteriologist Ogata Masanori.^72^ However, this theory was controversial and not uniformly accepted by governments and scientists around the world.^73^ As such, there was considerable disagreement as to what measures should be taken against rats on ships. Some scientists and bureaucrats viewed rats as a considerable risk. In 1899, British Indian naturalist E.H. Aitken wrote that he had often observed rats climbing up hawsers (cables) tying ships into docks, and passing ‘from boat to boat and from boat to shore with wonderful facility’. Aitken worried that they were spreading plague between ports in this way.^74^ Similar concerns were expressed in Australia a year later.^75^ Austro-Hungarian medical authorities, likewise, suspected that infected ship rats might play a role in the spread of plague through gnawing or soiling cargo, which could become ‘infected’ and spread the disease through supply lines if careful disinfection was not undertaken.^76^ Others, such as A. John Gregory, an Assistant Medical Officer of Health for the Cape Colony thought in 1900 that infected rats were the main danger and could be carried in cargo across the country.^77^

To avoid plague outbreaks, between 1899 and 1903, several states, cities, and ports began passing regulations that aimed at preventing rodents from swimming or climbing on or off board ships. The experts involved, whether sailors, zoologists, medical officials, or politicians, were largely convinced that one of the primary means by which rats boarded ships was via cables that connected ships to docks, or by swimming short distances. They thus started to employ architectural and spatial strategies to isolate all large vessels from the land to prevent rats from travelling between ship and shore. In attempting to curtail such ratty activities, humans began altering the spaces at which land and sea conjoined. In 1899 France, for example, the French Minister of the Interior mandated the inspection of ships in ports for rats, the protection of ‘mooring-cables … so as to prevent rats getting in or out of the ship by these means’, and the raising of footbridges at night. To kill any rats that snuck past such defences, he also dictated that ships should be fumigated with ‘sulphurous acid in all parts likely to harbour rats’.^78^ A few months later, in January 1900, the USA required all ships ‘infected or suspected of being infected with plague’ to be placed in ‘anchorage sufficiently remote from the nearest land or other vessel’ to prevent rats from swimming to land or to another ship.^79^

**Figure 2:**
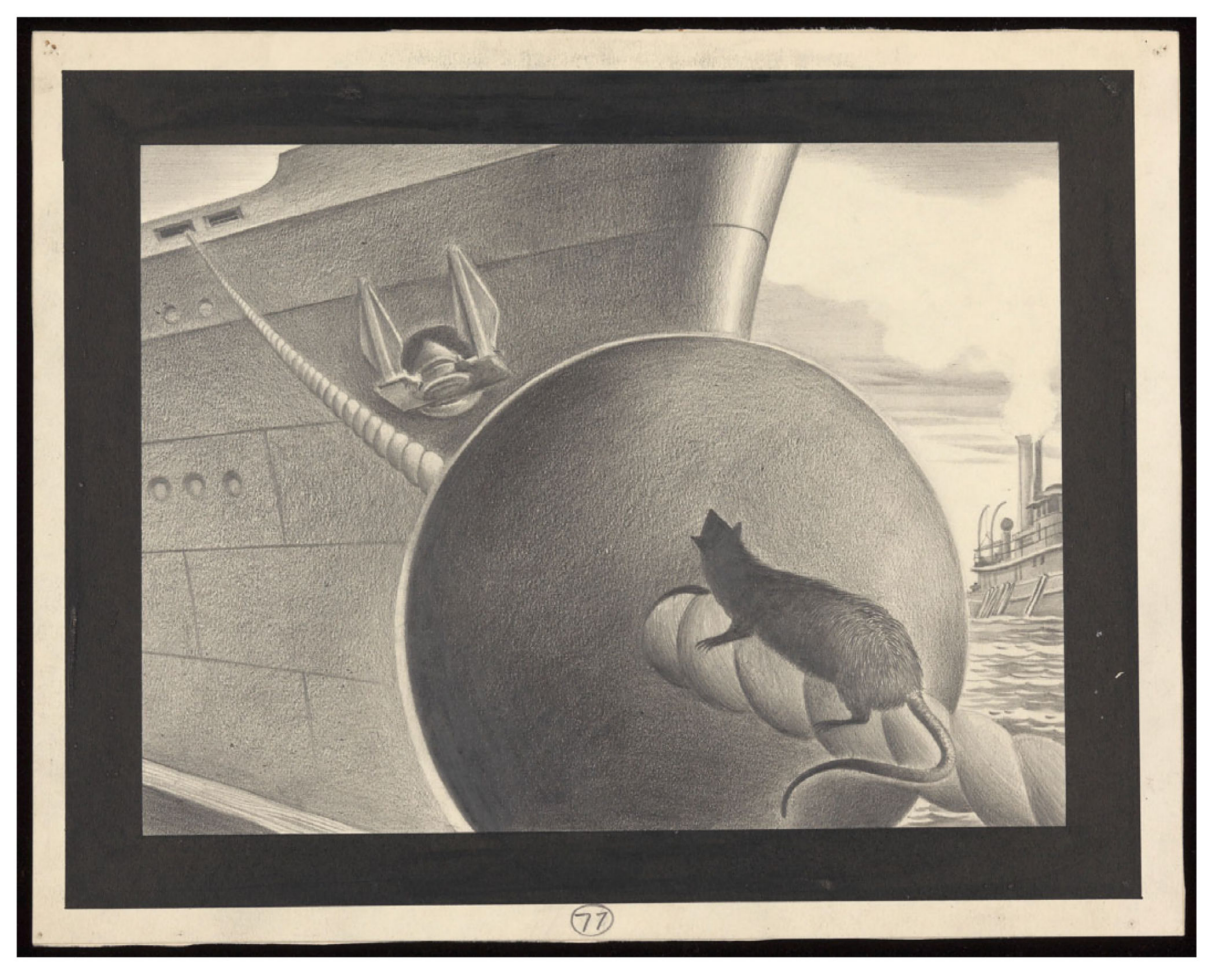
Illustration of a rat-guard barring a black rat from boarding a ship. ‘Obstructions on mooring-lines to stop rats boarding ships. Drawing by A.L. Tarter, 194-.’ Wellcome Collection: https://wellcomecollection.org/works/hcrv7jkv..

Other countries followed suit and began making use of an infrastructural strategy that had sometimes been used in Port Louis, Mauritius since the 1850s, and in the USA since the 1880s: the protection of hawsers with barriers that were later named ratguards (see Figure 2 for an illustration of what became a popular design). The early adoption of ratguards in Mauritius was hardly surprising: it had earned the moniker of an ‘island of rats’ in the eighteenth century.^80^ By the mid-nineteenth century, Port Louis was overrun with hordes of sugar-hungry rats climbing the cables that connected sea and shore by tethering ships to docks. To mitigate their ravages, officials began placing ‘boards’ that rats allegedly could not climb over – ‘on the mooring chains to prevent’ rats from embarking.^81^ The intent of such devices was simple: affix a tin-coated ‘circular piece of wood, like the head of a cask, made in two parts … on the cable at right angles’ to impede the path of rat and thus lock the animal on land or at sea.^82^ In 1888, a different design was patented in the USA by inventor Thomas Wilson: a funnel-shaped ‘protector for vessels against rats’.^83^ Here, rats could walk into the cone of the funnel, but purportedly not climb over it. Fearing the spread of plague, medical authorities in Odessa, then-Russia, began making use of these devices in 1899,^84^ and in Great Britain by April 1901.^85^ In the same year, the use of ‘metal funnel’ ‘sheathing’ devices on hawsers was mandated at the Table Bay Docks in the Cape Colony.^86^ In 1900, Australian authorities in Sydney and Melbourne mandated the use of ratguards, the tarring of mooring ropes, the raising and tarring of gangways at night, the docking of ships four feet from wharves and piers, and erected numerous other anti-rat defences.^87^ In some docks, they took these measures further, and declared war not only on ship rats, but also on land rats inhabiting wharves, so as to prevent plague from moving between seafarer and land-lubber rodents.^88^

Not all countries, however, were convinced of the rats’ role in spreading plague. British India, at the time the epicentre of the plague pandemic, was slow to adopt such measures, despite the insistence of some British Indian scientists, like Aitken, that rats circulated plague. Instead, its medical authorities remained largely convinced that rats played only a minor role in the spread of plague,^89^ even though bacteriological and observational evidence suggested that rats were susceptible to plague and could spread it on board or in dock.^90^ Accordingly, measures against rats were initially lax in parts of the country. According to O.V. Muller, a historian and political economist at Elphinstone College, anti-rat boundaries were not enforced in India’s largest port: Bombay. Ships were typically docked ‘up against the quay’, which allowed rats easily to swim or climb from sea to shore.^91^

From October to December 1903, an International Sanitary Conference was hosted in Paris where representatives from around the world passed international regulations to prevent the spread of plague. All signatories, encompassing the British Empire, the USA, and numerous European and Middle Eastern Nations agreed to a series of measures targeting rats on board ships.^92^ These encompassed inspecting unusual mortality among seafaring rats, killing rats on ships with cases of confirmed plague, and disinfecting any ‘infected’ merchandise.^93^ Additionally, the agreement empowered sanitary inspectors to issue certificates stating that rat destruction had been carried out.^94^ Fumigation was touted by delegates at this conference as the best available rat-destruction strategy, and was ultimately accepted as the eminent maritime disease control strategy.^95^ Two years later, the USA signed an agreement to similar effect with numerous South American countries.^96^ Rats on board ships were now internationally regarded as signs of potential contagion. Although it was considered near impossible to rid ships of their presence permanently, it was hoped that by stymieing their movements between ship and shore, the oceanic spread of plague could be prevented.

Following these agreements, nations around the world scrambled to pass local guidelines and regulations to prevent rats from crossing between shore and ship, and to kill those on board. By 1908, both anti-swimming strategies and ratguards were widely employed, although the specifications for their deployment varied from place to place.^97^ Yet both measures were met with problems. At times, closing the land/sea border to rats, meant also closing it to humans. In Bombay, 1905, the Health Officer of the Port, J. Sladen, found himself unable to comply with a request from the Australian Commonwealth to close the ‘openings in the side of a ship’ after sundown on ships with passengers on board, presumably because this would trap them on board overnight. Instead, he suggested that gangways between ship and dock should be ‘freshly tarred’ and either drawn up or ‘brightly illuminated and a watch kept on them’ at night, as was practice in Calcutta.^98^ On other occasions, such measures interfered too greatly with the flow of goods. By the end of 1905, in Aden, even keeping cargo boats and lighters ‘3 feet off jetty walls’ was deemed too difficult a logistical task, and the precautions were abandoned.^99^ In any case, the utility of a three feet rule was often in doubt. According to Dr G.A. Chitre, the author of an extensive report on the efficacy of measures imposed to prevent rats from boarding ships in Bombay, rats could potentially jump such a distance.^100^ Moreover, since the gangway undersides were never tarred, there was nothing to prevent rats from sneaking on board undetected in this manner.^101^

Rat’s climbing capabilities proved a tougher behaviour to curtail, and anti-climbing devices became a source of much controversy. A discussion of their efficacy in the Australian parliament in 1906 is a case in point. According to Billy Hughes, an MP for West Sydney, ratguards were so useless that ‘not only a rat, but a cow, could get ashore on the mooring ropes’. To make matters worse, their use was poorly enforced, meaning that on some ships tied up with five hawsers, only three or so would contain ratguards.^102^ Although other Australian government bureaucrats insisted that ratguards worked, the lived experience from sailors refuted such statements. Countering a claim from one MP that ‘rats cannot get over them [ratguards]’, politician James Matthews retorted: ‘sailors assure me that they do’.^103^ Ratguards were met with yet another problem in windy ports, such as Aden. In 1906, the Port Surgeon of Aden dismissed them as ‘useless’.^104^ The wires that affixed the guards to hawsers were too short to fasten them securely, which meant that they were liable to be blown out of position on windy days, and thus would have allowed rats to pass.^105^

A. John Gregory, the Medical Officer of Health for the Cape Colony was equally unconvinced by ratguards, and conducted an experiment which demonstrated, in a clear and simple manner, how utterly ineffective they were at impeding the movements of Cape rats between sea and shore. To simulate a docked ship, Gregory hung a cable over a tank of water, and attempted to prevent rats from crossing it. Firstly, he covered the cable with tar, which the rats crossed without any difficulty. Next, he placed a ‘large galvanised iron disk 18 inches in diameter’ in use in ‘Eastern ports’ on the rope.^106^ But the rats simply stood on their hind legs and vaulted themselves over. Finally, Gregory repeated his experiment with funnel guards, which he found equally pointless. Despite coating such funnels in grease, wire, or other materials, the rats ‘managed to negotiate every obstacle in the shape of disks’ up to a size of 20 inches, which he deemed impractically big for a guard. Nothing, apparently, could impede the progress of a determined rat without causing considerable disruption to shipping. And more importantly, rats were as likely to simply slip into cargo undetected.^107^

Between 1919 and 1921, similar experiments were conducted by Chitre in the Bombay Bacteriological Laboratory and observations made in the docks to the same conclusion. Chitre found that even if perfectly used, ratguards could never create an ‘absolute barrier, but only … a repellent to a certain extent’.^108^ To make matters worse, in a series of photographs, Chitre documented how poorly ratguards were enforced. Often, they were too small to be effective. At times they were placed so close to ships that rats could simply ignore them and jump aboard, and at other times they had large gaps between guard and rope, allowing rats to squeeze through.^109^ In short, human error and rodent cunning mitigated against efforts to prevent rats from climbing from docks to ships.

News of the inefficacy of these guards spread, and in the 1910 and 1920s, three new ratguard designs were devised. One, exhibited at the 1912 Philippine Exposition in Manila, which had been in use there for some four months, was a larger circular guard with additional reinforcement: a three foot long ‘galvanized-iron disk’ that was to be ‘held in place by four half tubes’ which extended out a foot in length.^110^ A second, somewhat different design was patented by American inventor F.E. Maynard in the same year.^111^ This ‘trapguard’ comprised a cylindrical disk, with a gate that a rat could easily push to enter. Maynard’s design arguably attempted to manipulate the sensory world of the rat to human advantage. Noting that the rat, confused by the presence of a barrier, would probably choose to enter the dark space, it was here that the coup de grâce would be delivered. Inside the guard was placed a ‘pad’ saturated with poison, which would coat the unsuspecting rat’s fur with poison, causing great tactile discomfort. Subsequently, the rats would clean themselves by licking off the poison, and thereby ingesting it.^112^ W.E. Rucker of the US Public Health and Marine Hospital Service endorsed this sadistic design as ‘very good’ and promised to display it at the ‘International Congress on Hygiene and Demography’.^113^ Just over a decade later, a third design, devised by Major J. Taylor of the Indian Medical Service along with Chitre, involved electrifying ‘half an inch parallel strips of aluminium on a hinged wooden triangular casing’ fastened around a hawser which would shock climbing rats and send them cascading to the ground. However, this design was ineffective in wet conditions, which caused the current to short circuit, allowing rats to cross ‘unharmed’.^114^ Despite their greater efficacy under certain conditions, all these new guards were rarely used: perhaps their more complex designs entailed additional expenses deemed beyond their utility.^115^

However, ratguards were faced with an additional problem: not only was their ability to prevent embarking and disembarking in doubt, they did nothing to assist in killing the rats living on board ships. They had to be paired with regular fumigation (typically with sulphuric acid) to kill the ship’s resident rodents, as well as the laying of poison in ports to catch escapees, and if any survived such measures, they could potentially re-establish large populations while at sea or on land.^116^ As noted by Engelmann and Lynteris, in the 1900s and 1910s, sulphuric fumigation as a rat-control strategy was not without its problems and was at times opposed by United States shipping companies.^117^ Similar complaints were expressed by British imperial companies in the 1920s. In 1922, Messrs. Turner Morrison and Company complained that fumigations’ utility was severely limited: rats simply escaped poisonous gases by running into ‘their homes behind casings of cabins and amongst wood work of upper structures’, where fumigants could not penetrate.^118^ In the same year, William Glen Liston, Director of the Bombay Bacteriological Institute, claimed that because each hold was usually fumigated separately, rats could escape death by moving around the ship. Moreover, storerooms could not be fumigated because the gases damaged food, and this could only be attempted after cargo was unloaded. Thus, the ‘most infectious place in the ships is usually passed over’.^119^

Ultimately, despite their inefficiency, these spatial and architectural strategies represented the best-known measures to curtail the perceived agency of rats in crossing between spheres of sea and shore. These pests and the pathogens they carried had exposed the porosity of sanitary borders between land and sea and forced humans to reconfigure how these spheres connected. Notwithstanding their inherent problems, ratguards, combined with more efficient fumigants such as hydrogen cyanide,^120^ remained the dominant strategies of preventing rats from exiting or boarding ships until 1925, when they were supplemented by a new infrastructural technique: the complete rat-proofing of vessels. Rat-proofers attempted not only to prevent the circulation of rats from ship to shore, but to transform the ship from a rodent paradise, replete with food, water, and nesting spaces, into a purgatory in which they could not meet their basic needs. In this way, rat-proofers moved beyond the fixation with rodents crossing between sea and shore and attempted to eliminate rodent eating, nesting, reproducing, and movement on board. Rat-proofing would ultimately succeed in transforming ships into no rats’ lands, thus producing new borders that greatly impeded the circulation of these animals between ports.

## Nesting and Movement: deconstructing and remaking borders

By the 1920s, rat-proofing had become perhaps the most important anti-rodent strategy in the terrestrial USA. Spurred by the success of an anti-plague rat-proofing campaign conducted in San Francisco between 1903 and 1908,^121^ which had eliminated plague from the city, numerous areas across the country were attempting to retrofit buildings to transform them from rat lodgings into architecturally hostile spaces.^122^ Their explicit goal was to bring about a permanent reduction in black and brown rat populations through building the rodents ‘out of existence’.^123^ This, ratproofing advocates argued, could be achieved by ridding buildings of all possible entrances and exists for rats, interspaces suitable for nesting, such as spaces between furniture and floor, or roof and ceiling, and eliminating runways which rats could use to access sources of food and water.^124^

With such successes in urban spaces, it was only a matter of time before shipbuilders began applying rat-proofing principals to vessels. The first experts to do so, were surgeon Samuel B. Grubbs and pharmacist Benjamin E. Holsendorf of the United States Public Health Service. In 1913, Grubbs and Holsendorf argued that fumigation ‘of vessels for rats could not be efficiently done unless the vessel was extensively prepared’. Such preparation, which involved ‘blocking runways by which rats could escape’, constituted a form of ‘partial rat-proofing’ from which a ‘more complete and permanent system’ could be developed.^125^ Hence, in 1924, they proposed a year-long experiment in total rat-proofing a ship, and ‘one of the finest and largest passenger vessels afloat was chosen for a demonstration’.^126^ While rat-proofing work was being carried out, near daily observations were taken and trapping operations conducted, and as the rat-proofing progressed, trapped rodents gradually fell to zero. By 1925, rats had successfully been built out of the ship.^127^

Following the successes of this demonstration, work commenced in 1925 on the rat-proofing of seventy-three ships of eighteen different lines,^128^ and Grubbs and Holsendorf publicised their methods in an eighteen-page illustrated paper in *Public Health Reports*. Their guidelines were aimed at large steamships, but could, in theory, have been applied to any ships. Rat-proofing entailed a fundamentally different approach to the anti-rodent borders of the 1890s–1920s, which in many ports remained in use into the 1940s despite their documented inefficacy.^129^ Instead of trying to prevent rats from crossing between spheres of land and sea, rat-proofers aimed to stop these animals from moving and breeding on ships, making it easy to kill them on board, and thus to prevent their migration between ports. In Grubbs and Holsendorf’s paper, rat-proofing was depicted as a process of spatial exclusion, in which the ship would be constructed or retrofitted to transform it into a no rats’ land. Its explicit goals, they argued, were to make it ‘impossible or difficult for a rat to hide, nest, or move about in search of food’.^130^ Essentially, it aimed to abolish all architectural features of ships that allowed rats to survive and thrive, transforming them into hostile environments in which rats would have access to little food, no suitable nests, and ferocious competition from their compatriots. In the words of Grubbs and Holsendorf, rats would be

confronted with an acute housing problem, high cost of living, and poor transportation between home and business (food getting). Labouring under these disadvantages, rats will be exposed to acute rivalry among themselves, to their enemies, and to starvation. They will breed with difficulty, and instead of multiplying will decrease or even disappear.^131^

**Figure 3:**
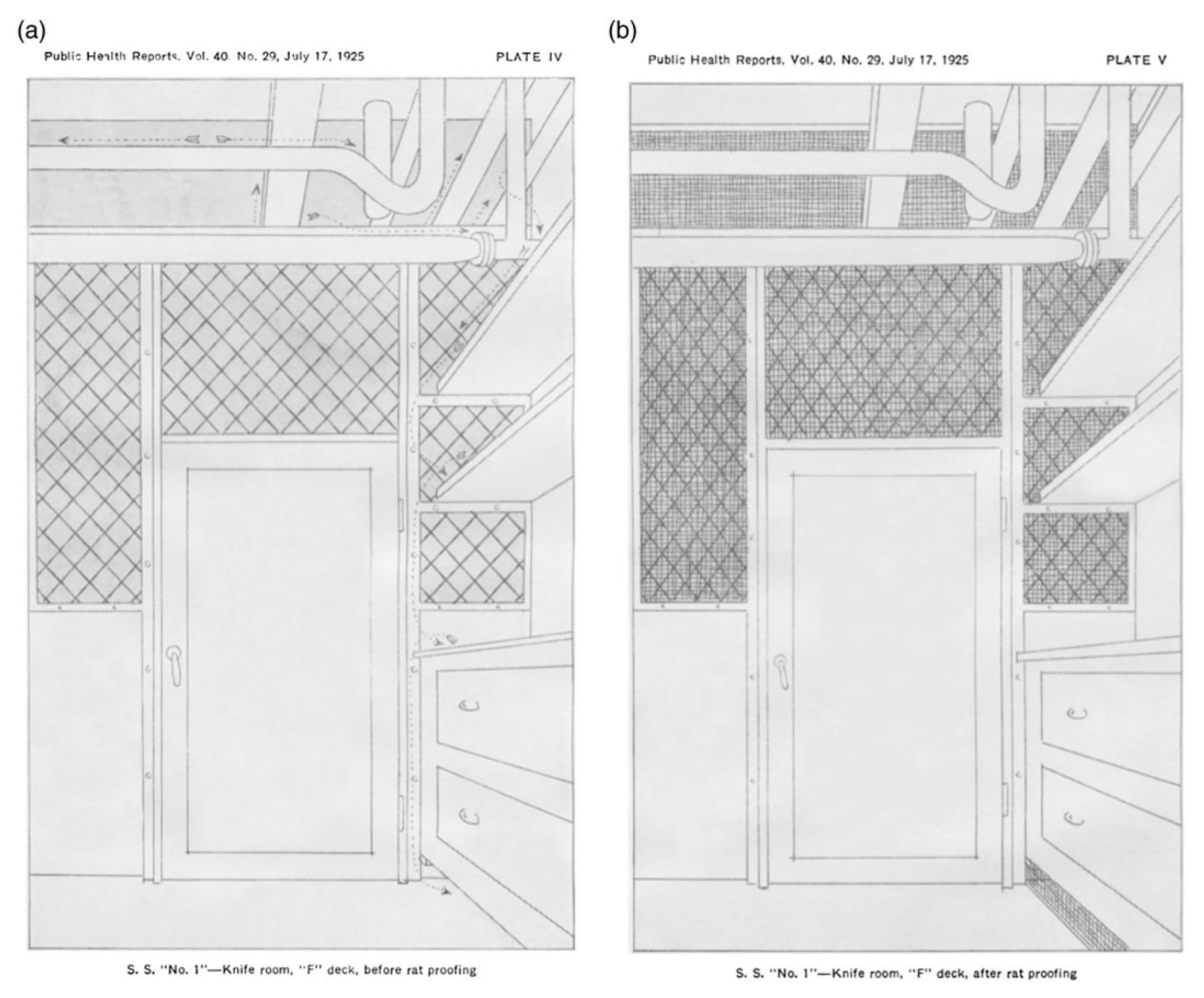
Plates IV and V from S.B. Grubbs and B.E. Holsendorf, ‘The Rat-Proofing of Vessels’, *Public Health Reports* 40, no. 29 (1925).

**Figure 4:**
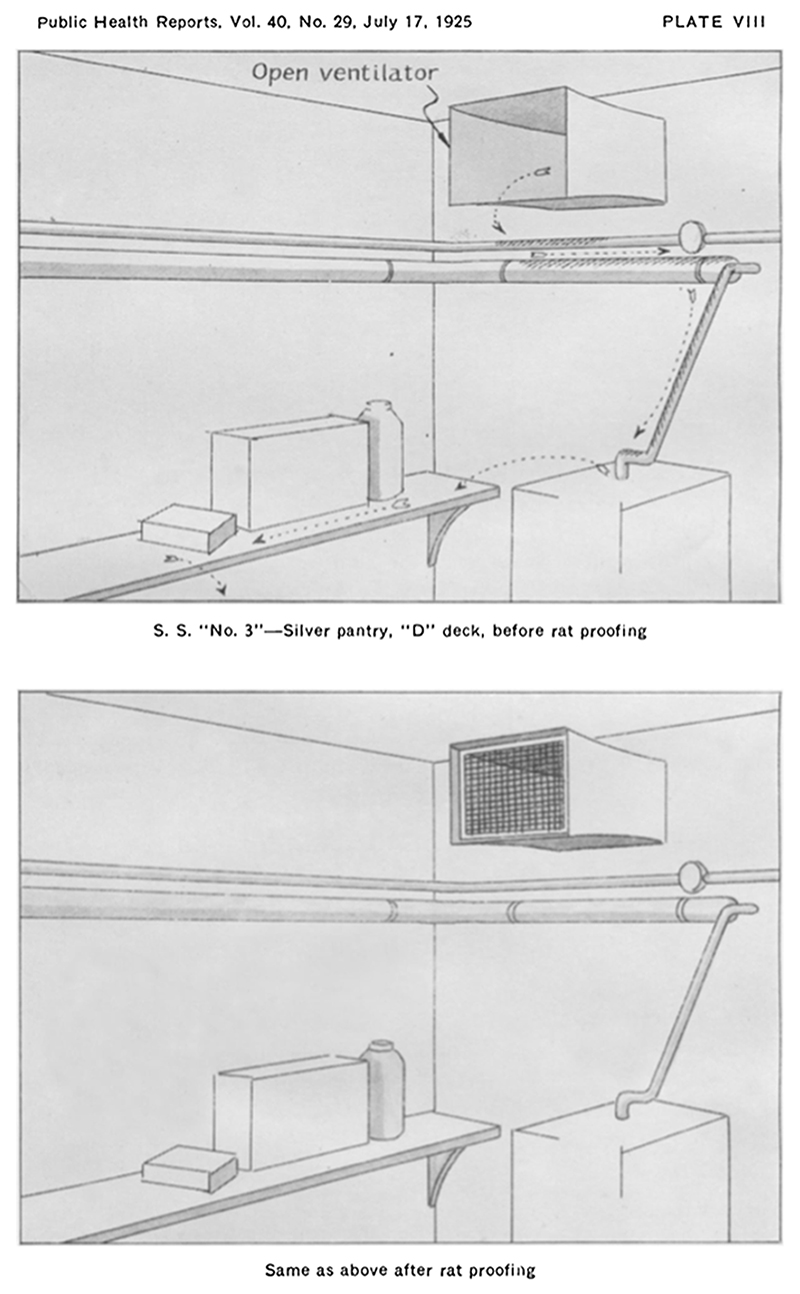
Plate VIII from S.B. Grubbs and B.E. Holsendorf, ‘The Rat-Proofing of Vessels’, *Public Health Reports* 40, no. 29 (1925).

Imagining the spatial and sensory world of rats was key to the success of rat-proofing. Building upon knowledge produced by ratcatchers and fumigators about the sensorial and spatial worlds of ship-rats, rat-proofers attempted to end the unrestricted rodent movement that so violated the rigid spatial ordering of life at sea.^132^ To achieve a rat-free maritime world, rat-proofers needed to think like rats to deprive them of their basic needs. To prevent them from gnawing their way into produce containers, nibbling at garbage, or chewing holes in water tanks and casks, one could place goods in rat-proof containers, or in rooms ‘devoid of rat harbourage and into which rats can not come from without’.^133^ To impede their climbing and scurrying in search of food, they could place screens ‘impenetrable to rats at gnawing levels’ over doors and windows.^134^ To deprive them of cosy, secluded and dark spots in which to nest, one needed to locate gaps between furniture and the floor, secluded openings of ventilation shafts, or spaces between decks, and block them with ‘material impenetrable to rats’.^135^ The process is perhaps best illustrated by the following ‘before and after’ diagrams:

In the before illustration (Figure 3, Plate IV), we can see the imagined or observed path of the rat as illustrated by arrows. The diagram argues that this room, before rat-proofing, provides many highways for rats, who could climb along the piping in and out of the room, up and down the shelves, and hide underneath the chest of drawers. By contrast, in the ‘after’ illustration (Figure 3, Plate V), whilst rats could probably still climb around the room, they were deprived of both hiding places and paths between this room and the rest of the ship, thanks to the installation of screens over all openings. Likewise, in Grubbs and Holsendorf’s diagram of a pantry (Figure 4), in the ‘before’ image, the rat can scurry in through the vent, access all the food, and then move on or escape through the vent once again. By closing the vent in the ‘after’ photograph, the rat is subsequently deprived of access to food altogether.

Applying these practices required considerable expertise because the spatial and sensory worlds of rats and humans were so vastly different. The geography of the ship had to be considered not from a human perspective, but via a rats-eye-view. According to C.L. Williams, humans were accustomed to looking ahead, or down, and rarely above. ‘Runways’, he argued

may be anywhere, but the best place to look for them is where one would never think of looking. The truth of this paradoxical statement may readily be verified by placing an experienced inspector and a neophyte on the same ship. The former will soon have spotted every run on the ship; the latter will take a month to find them all. This is partly due to man’s instinct to direct his eyes to the ground, while the runways are mostly overhead … ^136^

Such advice, along with instructional photographs and diagrams produced by the US Public Health Service visually reconfigured spaces once completely innocuous, or even useful to sailors, as potential rodent homes.^137^ Furnishings, building materials, and goods were constructed as pathological objects and reframed in terms of their uses to rats. Gaps between fittings, unenclosed storage areas, and rooms without netting were transformed into ‘harbourage’ that rats might contaminate. Piping, tightropes, or shelves were recast as rat ‘runways’ or ‘highways’ through which they might move between parts of the ship, encounter other rats, breed, and risk infecting sailors. Scrap material or stored possessions were depicted as nesting materials.

Through accessing the spatial world of the rat, rat-proofers developed one of the most successful anti-rodent measures yet attempted. According to Grubbs, total rat-proofing was quickly becoming a more efficient rodent control strategy than fumigation. In 1927, he reported that in contrast to fumigation, which was only 70–80% effective,^138^ and temporary in effect,^139^ total rat-proofing gave permanent results. As evidence of its efficacy, Grubbs noted that it had quickly become a popular measure and was widely used by New York shipping businesses, as well as within the United States Navy and Army Transport Service vessels.^140^ According to C.L. Williams, it was so effective that even in the case of one ‘vessel engaged in the West Indies trade’, which had harboured an average population of 1177 rats, rat-proofing had gradually reduced that number to zero.^141^ The wholesale exclusion of rats from vessels was now believed to be a possibility.

Total rat-proofing appears to have been widely accepted as a key tool in the maritime war against rats. Yet not everyone was convinced that it should be used to evict these animals entirely. James Alexander Mitchell, the Secretary of Public Health of South Africa, thought that rat-proofing should be employed on ships, but only to limit the spaces accessible to rats. A few individuals, in his opinion, could serve as an early warning system for plague, much like canaries in coalmines. Rats, he argued should be offered their own ‘special chambers or spaces … where they could find attractive cover and food, but which could be easily shut off when desired’.^142^ By keeping these rats in a secure and bounded space, they could act as ‘sentinels’ or ‘detectors of plague infection’.^143^ In other words, once these rats got infected, the crew would know that plague was on board. Mitchell’s suggestion that rats might yet serve some purpose on board rat-proof ships appears to have been unpopular or overlooked. Although this article was published in a British journal and reprinted in French,^144^ there is no evidence that Mitchell’s strategy was ever used outside of South Africa and at the time of publication, Mitchell had not yet tested it on ships himself.^145^

Instead, shipbuilders and engineers in North American and some British shipyards continued to apply Grubbs and Holsendorf’s principals in pursuit of a utopian ideal of eliminating seafarers’ pestilent companions from ships entirely. According to Vernon B. Link’s survey of the US Public Health Service’s Annual Reports, shipping companies quickly began experimenting with rat-proof construction. By 1926, rat-proofing of ships had commenced in ‘Southampton, Bremen, Danzig, Buenos Aires, Gothenburg and Bergen’.^146^ The rapid adoption of these techniques likely owes much to Grubbs and Holsendorf’s 1926 manual, *The Rat-Proofing of Vessels* which was translated into multiple languages and had gone through three editions by 1931.^147^ In this booklet, the authors strongly pushed an economic argument: rat-proof construction was a cost-saving device. Not only could ships be rat-proofed at minimal cost, but their role in keeping rat populations to an absolute minimum would prevent damage to cargo and lessen the need for fumigation along with the shipping delays it caused.^148^ Fumigation, once promoted as a measure to *avoid* shipping delays,^149^ was now being attacked as a *source* of further delays by rat-proofers. At this point, avoiding routine fumigation through rat-proofing had become a possibility following the International Sanitary Convention of Paris in 1926, which empowered port health officers to issue ‘deratisation’ exemption certificates. When investigating ships for rats, inspectors paid particular attention to rat harbourage and exemptions could be issued if an official was ‘satisfied that the ship is maintained in such a condition that the rat population is reduced to a minimum’.^150^ By 1929, American Marine Standards Guidelines recommended that all shipowners take steps to rat-proof their vessels,^151^ and although these were not mandated, all ships funded by any money loaned from the United States Government had to comply to such regulations.^152^

This combination of financial incentive and government regulations meant that rat-proof ships sailed widely across the globe by the early 1930s: as many as 75% of the ‘better-class ships’ coming into New York from 47 companies and 14 nations from the Americas, Europe, South America, and Asia had been rat-proofed.^153^ Thanks to such successes in rat-proofing, in May 1937, the Quarantine Commission of the *Office International D’Hygiene Publique*, which consisted of representatives from the French, British, German, Belgian, Dutch, and American empires, confirmed that sanitary authorities could neglect the ‘deratisation’ of rat-proofed parts of the ship, and still receive an internationally-valid certificate stating that deratisation had taken place.^154^ In the same year, an article in *Public Health Reports* declared that the overseas transmission of plague was ‘A Danger Almost Eliminated’.^155^ A study of 4,418 ships at ‘Atlantic ports between July 1, 1936 and January 31, 1937’, revealed that only 8.4% of ships were infested with rats, in comparison to 50% a decade earlier. This, the author attributed to a combination of rat-proofing, more effective fumigation, and intensive inspections of vessels.^156^ In 1948, G.L. Dunnahoo, a United States quarantine official reported that numerous shipbuilding companies had found that ratproof ships required ‘less tons of steel and less man hours’ to produce relative to other ship designs. As a result of this, rat-proofing bloomed in popularity, and in United States ports, the number of fumigations was ‘steadily dropping off’ and correspondingly, so were shipping delays.^157^ Just a few years later Kund Stowman, a US Delegate to the Special Committee of the World Health Organisation on the International Sanitary Regulations, claimed that ‘a large majority of ocean going ships’ had been rat-proofed, the result of which was that deratting operations were becoming increasingly unnecessary.^158^ Rats, the unwanted but constant animal companions of humans on ships for hundreds of years had, in many cases, now been built out of maritime spaces.

Ultimately rat-proofers, although able to prevent rats from making permanent homes in ships, abandoned the fantasy that anti-rat borders between land and sea could be clearly delineated and policed by humans, even in cases where ratguards were still in use. According to C.V. Akin, Chief Quarantine Officer of New York, the term ‘ratproofing’ itself was somewhat of a misnomer. In ‘the natural course of events’, he argued, ‘any vessel, regardless of the way in which it has been built, may pick up rats in cargo or rats may come on board by way of ship lines and gangways’. With rat proofing, rats were now unable to ‘roam at will’ and could ‘be cornered and eliminated by trapping or by other direct combat measures’,^159^ and thus prevented from disembarking. Essentially, in transforming the ship into a no rats’ land, rat-proofers created a new border which impeded rodent movements between ports. Thus, by the 1930s, in response to the movements, habits, and settlement practices of rats, humans had not only reshaped boundaries between sea and shore, but also transformed the vessels that moved between them into anti-rodent barriers in their own right.

## Conclusion

Rats left their marks on maritime architecture, and on international trade and transport in the nineteenth and twentieth centuries. Such traces were not only physical; ideas about what rats might do to humans, domestic animals, cargo, and boats themselves haunted the designs of ships and docks long after rats had stopped devouring sails. At sea, rats obstructed international trade, ate away at capitalist coffers, damaged property, disrupted imperial knowledge networks, and unwittingly facilitated the global spread of plague.

As knowledge about rats changed, so too did the spaces and objects that connected spheres of land and sea. In the nineteenth century, rats climbed gangways and hawsers which tethered ships to shore with few impediments or found themselves imported into cargo holds. Seafarers generally viewed them as an unavoidable part of maritime life, and once on board, rats engaged with humans in a variety of ways. Some who were accustomed to a more segregated hierarchy of life saw rats as abominable pests and complained of their invasions from the lower quarters into their own. Others took umbrage at their attacks on food, cargo, and flesh. Several seafarers, however, contended that rats were harmless, and tried to form cordial relationships with them, hunted them for sport, or consumed them as food or medicine.

In the wake of the Third Plague Pandemic, and the gradual acceptance of rats being the primary agents in the spread of plague, the fundamentals of human-rodent relations on ships began to change. As the rat was transposed into a pathogenic object, the variegated multispecies relationships on board became more monochrome. Rats were no longer sources of food which might ward off hunger or scurvy; they no longer served as on-board companions, or signs of good luck, eternally present on ships, regardless of human attempts to control them. Instead, as carriers and spreaders of plague, rats were now the greatest animal ‘enemies of man’; they were no longer permitted to hitch a ride on, nor make their homes in, the bowels of the ships that kept the global economy moving. Accordingly, various scientists, government officials, and inventors constructed an anti-rodent border which aimed to block the throughfares that connected ship to shore, and thus to prevent rats from swimming or climbing on board.

However, such a border proved an unrealisable ideal: rats were resourceful and learned to circumvent the infrastructural innovations. This strategy also neglected the fact that ships were perfect habitats for rats, which the animals rarely wanted to leave, and in which the survivors of fumigation could raise new families. To address this problem, rat-proofers developed new infrastructure to prevent nesting, breeding, and movement on board. To achieve this, they reframed the architecture of the ship in terms of the rats’ own spatial and sensory perceptions. Rather than viewing the ship as a series of rigidly separated series of spaces for cargo, food, water, and humans of various classes, they imagined it as a multitude of potential rat habitats that needed to be enclosed, covered, and plugged. Gaps between fittings, along with shelving and piping were reconceived as ‘highways’ through which rodents could move. Dark spaces underneath furniture or between the decks were damned as ‘harbourage’ in which rats might nest. Objects stored outside of containers were declared potential nesting materials. In building rats out of the ship, rat-proofers succeeded in erecting a new anti-rat barrier: the ship as a no-rats’ land, and a border between ports.

Reflecting on the history of these scurrying seafarers ultimately shows how fragile boundaries between land and sea have been in the past. These boundaries were fluid and shifted in response to a multitude of interactions between rats, humans, ships, hawsers, ratguards, cargo, fleas, and *Yersinia pestis*. Despite the attempts of humans to exclude rats from oceanic crossings, these terraqueous animals continued to move between spaces separating ships and land, hinterland and docks, ports, and oceans. Only the comprehensive reconstruction and retrofitting of ships succeeded in greatly limiting, but never fully eliminating, the maritime movement of rats.

## Footnotes

1 United States Public Health Service, *The Ship’s Medicine Chest and First Aid at Sea* (Washington: Government Printing Office, 1955), 380.

2 Jari Ojala and Stig Tenold, ‘Maritime Trade and Merchant Shipping: The Shipping/Trade Ratio since the 1870s’, *International Journal of Maritime History* 29, no. 4 (1 November 2017): 838–54; Sujit Sivasundaram, *Waves across the South: A New History of Revolution and Empire* (Chicago: The University of Chicago Press, 2020); Gwyn Campbell, *Africa and the Indian Ocean World from Early Times to circa 1900*, 2019, 178; Michael N. Pearson, *The Indian Ocean* (London; New York: Routledge, 2003), 191; S. G Sturmey, *British Shipping and World Competition* (Oxford: Oxford University Press, 2017), 1–4.

3 See for example: Robert A Kilmarx, *America’s Maritime Legacy: A History of the U.S. Merchant Marine and Shipbuilding Industry since Colonial Times* (Boulder, Colo.: Westview Press, 1979); Ojala and Tenold, ‘Maritime Trade and Merchant Shipping: The Shipping/Trade Ratio since the 1870s’.

4 Kaori Nagai, ‘Vermin Writing’, *Journal for Maritime Research* 22, no. 1–2 (2 July 2020): 59.

5 Lucinda Cole, *Imperfect Creatures* (University of Michigan Press, 2016), Chapter 5; See also Alfred Crosby, *The Columbian Exchange: Biological and Cultural Consequences of 1492*, 30th Anniversary Edition (Westport, Connecticut: Praeger, 2003), 97; McNeill, John Robert, ‘Of Rats and Men: A Synoptic Environmental History of the Island Pacific’, *Journal of World History* 5, no. 2 (1994): 299–349.

6 Cole, *Imperfect Creatures*, 39.

7 Ibid., 144.

8 Nagai, ‘Vermin Writing’.

9 Beyond a few references to vermin or livestock on board, or marine animals sighted in the sea. For example: Michael N. Pearson, *The Indian Ocean* (London; New York: Routledge, 2003), 207; David Hastings, *Over the Mountains of the Sea: Life on the Migrant Ships 1870–1885*. (New York: Auckland University Press, 2013), Chapters 1 & 2; Tamson Pietsch, ‘Bodies at Sea: Travelling to Australia in the Age of Sail’, *Journal of Global History* 11 (2016): 220.

10 Lukas Engelmann and Christos Lynteris, *Sulphuric Utopias: A History of Maritime Fumigation* (Cambridge, Massachusetts: The MIT Press, 2020).

11 Sari Mäenpää, ‘Sailors and Their Pets: Men and Their Companion Animals Aboard Early Twentieth-Century Finnish Sailing Ships’, *International Journal of Maritime History* 28, no. 3 (1 August 2016): 480–95.

12 Derek Lee Nelson, ‘The Ravages of Teredo: The Rise and Fall of Shipworm in US History, 1860–1940’, *Environmental History* 21, no. 1 (1 January 2016): 100–124; Nagai, ‘Vermin Writing’.

13 Martha Chaiklin, Philip Gooding, and Gwyn Campbell, *Animal Trade Histories in the Indian Ocean World*, 2020.

14 Pamila Gupta, Isabel Hofmeyr, and M. N. Pearson, eds., ‘The Idea of the Ocean’, in *Eyes across the Water: Navigating the Indian Ocean*, 1st ed, Indian Ocean Series (Pretoria: Unisa Press, 2010), 8–9; Edward A. Alpers, *The Indian Ocean in World History*, New Oxford World History (Oxford; New York: Oxford University Press, 2014), 5–7; Michael N. Pearson, ‘Intorduction: Maritime History and the Indian Ocean World’, in *Trade, Circulation, and Flow in the Indian Ocean World*, ed. Michael N. Pearson, Palgrave Series in Indian Ocean World Studies (Houndmills: Palgrave Macmillan, 2015), 2–3.

15 Rainer F. Buschmann, ‘Oceans of World History: Delineating Aquacentric Notions in the Global Past1’, *History Compass* 2, no. 1 (1 January 2004): 1; Michael S. Reidy, *Tides of History: Ocean Science and Her Majesty’s Navy* (Chicago: University of Chicago Press, 2008), 10; Rila Mukherjee, ‘Escape from Terracentrism: Writing a Water History’, *Indian Historical Review* 41, no. 1 (1 June 2014): 87–101.

16 Nigel Worden, ‘Writing the Global Indian Ocean’, *Journal of Global History* 12, no. 1 (2017): 145–54; Shanti Moorthy and Ashraf Jamal, ‘Introduction: New Conjunctures in Maritime Imaginaries’, in *Indian Ocean Studies: Cultural, Social, and Political Perspectives*, ed. Shanti Moorthy and Ashraf Jamal, Routledge Indian Ocean Series 6 (New York: Routledge, 2010), 4; Ralph Callebert, *On Durban’s Docks: Zulu Workers, Rural Households, Global Labor*, Rochester Studies in African History and the Diaspora, v. 76 (Rochester: University of Rochester Press, 2017).

17 Wilko von Hardenberg, ‘Knowing the Littoral: Perception and Representation of Terraqueous Spaces in a Global Perspective’, *Isis* 112, no. 1 (2021): 108.

18 For an analysis of how such connections are constructed historically through visual materials see Sujit Sivasundaram, ‘Towards a Critical History of Connection: The Port of Colombo, the Geographical “Circuit,” and the Visual Politics of New Imperialism, ca. 1880–1914’, *Comparative Studies in Society and History* 59, no. 2 (2017): 346–84.

19 This concept is similar to Michael Pearson’s (2003) assertion of the need to think “amphibiously” in Indian Ocean History. However, terraqueous is a more useful term as it lacks the biological connotations associated with amphibious animals. Pearson, *The Indian Ocean*, 5, 30; Alison Bashford, ‘Terraqueous Histories’, *Historical Journal* 60, no. 2 (2017): 255.

20 Bashford, ‘Terraqueous Histories’, 272.

21 Black rats have been recorded as swimming from ships to shore as far as 500 metres, while brown rats can swim a remark-able 2 kilometres in ideal conditions. See J.C. Russell and M.N. Clout, ‘Rodent Incursions on New Zealand Islands’, in *Proceedings of the 13th Australasian Vertebrate Pest Conference* (Lincoln, New Zealand: Landcare Research, 2005), 325; John P. Parkes, Andrea E. Byrom, and Kerri-Anne Edge, ‘Eradicating Mammals on New Zealand Island Reserves’, *New Zealand Journal of Ecology* 41, no. 2 (2017): 264.

22 Campbell argues that environments, climates, and rain patterns in the Indian Ocean World “did not respect man-made political boundaries” but stops short of including animals within this discussion. Gwyn Campbell, *Africa and the Indian Ocean World from Early Times to circa 1900* (Cambridge: Cambridge University Press, 2019), 17.

23 Adam McKeown, ‘A World Made Many: Integration and Segregation in Global Migration, 1940–1940’, in *Connecting Seas and Connected Ocean Rims: Indian, Atlantic, and Pacific Oceans and China Seas Migrations from the 1830s to the 1930s*, eds. Donna Gabaccia and Dirk Hoerder (Leiden: Brill, 2011), 42–64; Harriet Ritvo, ‘President’s Lecture Going Forth and Multiplying: Animal Acclimatization and Invasion’, *Environmental History* 17, no. 2 (1 April 2012): 404–14; Pearson, *The Indian Ocean*, 193; Reidy, *Tides of History*, 274.

24 Engelmann and Lynteris, *Sulphuric Utopias*.

25 T.W. Higginson, ‘Our Menagerie. IV.–Rats’, *Our Young Folks: An Illustrated Magazine for Boys and Girls* 6, no. 8 (1870): 488.

26 Oxford English Dictionary, ‘“Rodentia, n.”.’, n.d., https://www.oed.com/view/Entry/275785?redirectedFrom=rodentia; Oxford English Dictionary, ‘“Rodent, Adj. and n.”.’, n.d., https://www.oed.com/view/Entry/166814.

27 Nagai, ‘Vermin Writing’, 61.

28 Italics original. Henry Juta, *Reports of Cases Decided in the Supreme Court of the Cape of Good Hope During the Years 1885–6* (Cape Town: J.C. Juta & Co, 1887), 53.

29 Nagai, ‘Vermin Writing’, 65–66.

30 ’Editorial’, *Shipping and Mercantile Gazette*, 23 October 1875, 5.

31 Nagai doubts that rats were responsible for damaging the timbers, suspecting that white ants and shipworms were the culprits. She argues that the “famed destructiveness of rats” (65) was a convenient scapegoat for human error, or the agency of other verminous animals in ships. Nagai, ‘Vermin Writing’, 63–65.

32 Ibid., 61.

33 Even a vague date is not specified by the author.

34 L.R.C.P., ‘Rats on Board Ships’, *The Nautical Magazine* 44, no. 12 (1875): 983.

35 Hastings, *Over the Mountains of the Sea*, Introduction; Pearson, *The Indian Ocean*, 234; Pietsch, ‘Bodies at Sea: Travelling to Australia in the Age of Sail’.

36 Hastings, *Over the Mountains of the Sea*, Chapter 1; Greg Dening, *Mr Bligh’s Bad Language: Passion, Power and Theatre on the Bounty*, Canto (Cambridge: Cambridge Univ. Press, 1994), 19–24.

37 Dening, *Mr Bligh’s Bad Language*, 19–24; Frances Steel, ‘Anglo-Worlds in Transit: Connections and Frictions across the Pacific’, *Journal of Global History* 11 (2016): 264–70; William Hasty and Kimberley Peters, ‘The Ship in Geography and the Geographies of Ships’, *Geography Compass* 6, no. 11 (2012): 663–68; Andrew Davies, ‘From “Landsman” to “Seaman”? Colonial Discipline, Organisation and Resistance in the Royal Indian Navy, 1946’, *Social & Cultural Geography* 14, no. 8 (2013): 871; Michael Pearson, ‘Places in the Indian Ocean World’, *Journal of Indian Ocean World Studies* 1 (2017): 5–11; Sarah Crabtree, ‘Navigating Mobility: Gender, Class, and Space at Sea, 1760–1810’, *Eighteenth-Century Studies* 48, no. 1 (2014): 102; Pearson, *The Indian Ocean*, 209; Frances Steel and Manchester University Press, *Oceania under Steam: Sea Transport and the Cultures of Colonialism, c.1870–1914*, 2017, Chapter 3.

38 For example see: Johanna de Schmidt, ‘“This Strange Little Floating World of Ours”: Shipboard Periodicals and Community-Building in the “Global” Nineteenth Century’, *Journal of Global History* 11 (2016): 242.

39 Thomas Boteler, *Narrative of a Voyage of Discovery to Africa and Arabia Performed by His Majesty’s Ships Leven and Barracouta, From 1821 to 1826, under the Command of Capt. F.W. Owen, R.N*. (London: Samuel Bentley, 1835), 474; R.S., ‘Smoking Ship’, *Gentleman’s Journal*, 1 March 1870, 320.

40 L.R.C.P., ‘Rats on Board Ships’, 983.

41 Boteler, *Narrative of a Voyage of Discovery*, 474–75.

42 Francis Buckland, *Curiosities of Natural History* (New York: Follett, Foster & Co, 1864), 182.

43 Sinclair Thomas Duncan, *Journal of a Voyage to Australia by the Cape of Good Hope* (Edinburgh: Reid and Reynolds, 1869), 158.

44 *Instructions from the Commanders of the East-India Company’s Own Ships to Their Officers, &c*. (London: E. Cox and Son, 1819), Appendix, 44; Lemuel Norton, *Auto-Biography of Lemuel Norton* (Portland: Advertiser Steam Job Printing Office, 1861), 36; George Barrell, *Notes of Voyages and Incidents Connected Therewith in a Career of Thirty Years at Sea, with Other Miscellaneous Matter* (Springfield, Ill.: H.W. Rokker, Printer and Binder, 1890), 141–42; James Gordon, ‘Art IX.-A Pilgrim Voyage in the 19th Century’, *Calcutta Review* 109, no. 215 (1899): 101; For rats, boundary crossing and underclasses, see Lisa T. Sarasohn, *Getting under Our Skin: The Cultural and Social History of Vermin* (Baltimore: Johns Hopkins University Press, 2021), Chapters 8–9.

45 Nagai argues that rats were so ubiquitous on ships that often their presence in ships logs and other materials was ‘not worthy of remark’ and thus did not make their way into ‘sailors’ journals. It is possible that seasoned ‘lower class’ sailors were well accustomed to rats, hence the lack of such material. Nagai, ‘Vermin Writing’, 60.

46 Charles Reade and Dion Boucicault, ‘Foul Play’, *Once a Week* 1, no. 3 (18 January 1868): 56; Harriet Beecher Stowe, *Uncle Tom’s Cabin; or, Life Among the Lowly* (Boston: John P. Jewett & Company, 1852), 258; Frances Stoughton Bailey, *Story and Song* (Providence: Snow & Farnham, 1894), 74.

47 Ralph Watts Leyland, *A Holiday in South Africa* (London and Liverpool: Sampson Low, Marston, Searle, and Rivington, 1882), 314. Nagai notes that this was a common complaint of seafarers. Nagai, ‘Vermin Writing’, 68.

48 R.S., ‘Smoking Ship’, 320.

49 Vermin may refer not only to rats, but also cockroaches and other insects. ‘Captured Slave Ship, Jamaica, 1857’, *Slavery Images: A Visual Record of the African Slave Trade and Slave Life in the Early African Diaspora*, http://www.slaveryimages.org/s/slaveryimages/item/2740. The original article cited is ‘Capture of a Slaver’, *The Illustrated London News*, 11 May 1857.

50 According to James Walvin, citing slave trader John Newton, rats nibbled ‘at the flesh of sleeping Africans’, James Walvin, *Crossings: Africa, the Americas and the Atlantic Slave Trade* (London: Reaktion Books, 2013), 96.

51 From the documentation available in published primary sources (as well as some manuscripts), I found only two examples of blocking points of entry to rats in this century, in Port Louis and London. By contrast, there are dozens of accounts of rat-killing strategies. T.E. Palmer, G.T. Bradshaw, and G. Clark, ‘*The Mauritius Register: Historical, Official & Commercial, Corrected to the 30th June 1859*’ (Mauritius: L. Channell, 1859), lxiii; Andrew Wynter, *Fruit Between the Leaves*, vol. 1 (London: Chapman and Hall, 1875), 209.

52 Nagai, ‘Vermin Writing’, 65.

53 ’Varmint’, *The New Sporting Magazine* 58 (1869): 88.

54 Nagai, ‘Vermin Writing’, 65.

55 Ibid., 65; James Rodwell, *The Rat: Its History & Destructive Character* (London: Routledge & Co, 1858), 163.

56 Robert White Stevens, *On the Stowage of Ships and Their Cargoes* (London: Longmans, Green and Company, 1867), 597.

57 Higginson, ‘Our Menagerie. IV.–Rats’, 488.

58 Elisha Kent Kane, *Arctic Explorations: The Second Grinnell Expedition* (Philadelphia: Childs and Peterson, 1856), 394.

59 Nagai, ‘Vermin Writing’, 62–63.

60 L.R.C.P., ‘Rats on Board Ships’, 982.

61 R.S., ‘Smoking Ship’, 321.

62 Buckland, *Curiosities of Natural History*, 182.

63 Ibid., 181–82.

64 Nagai, ‘Vermin Writing’, 70; Hastings, *Over the Mountains of the Sea*, Chapter 9.

65 For example, in Henry Mayhew’s interviews with rat-catchers, rats are not associated with diseases. Neil Pemberton, ‘The Rat-Catcher’s Prank: Interspecies Cunningness and Scavenging in Henry Mayhew’s London’, *Journal of Victorian Culture* 19, no. 4 (2 October 2014): 531–32; See also Engelmann and Lynteris, *Sulphuric Utopias*, 82–83.

66 Quote on p 141 Barrell, *Notes of Voyages and Incidents Connected Therewith in a Career of Thirty Years at Sea, with Other Miscellaneous Matter*, 141–42.

67 Kane, *Arctic Explorations*, 395.

68 Myron Echenberg, *Plague Ports: The Global Urban Impact of Bubonic Plague, 1894–1901* (New York: NYU Press, 2010).

69 Christos Lynteris, *Ethnographic Plague: Configuring Disease on the Chinese-Russian Frontier* (London: Palgrave Macmillan, 2016), 1.

70 David S. Barnes, ‘Cargo, “Infection,” and the Logic of Quarantine in the Nineteenth Century’, *Bulletin of the History of Medicine* 88, no. 1 (2014): 75–101; Alison Bashford, *Quarantine: Local and Global Histories* (Basingstoke, Hampshire: Palgrave Macmillan, 2016); Engelmann and Lynteris, *Sulphuric Utopias*, chap. Introduction.

71 A detailed examination of how ship-rats specifically became associated with disease (rather than rats in general) is beyond the scope of this paper and will be the subject of a future work. For an examination of the relationship between rats and maritime disease control in this period, see Engelmann and Lynteris, *Sulphuric Utopias*, Chapter 3.

72 Masanori Ogata. ‘Über die Pestepidemie in Formosa’, *Centralbl. f. Bakteriol*. 1897; 21:769–77.

73 According to Engelmann and Lynteris, only in 1906 was this theory ‘fully accepted’. Engelmann and Lynteris, *Sulphuric Utopias*, 84.

74 E.H. Aitken, ‘Rats and the Plague’, *The Times of India*, 19 July 1899, 4.

75 Frank Tidswell, ‘On Plague and Its Dissemination: Address Delivered Before the N.S.W. Branch of the British Medical Association, 27 April 1900’, *Australasian Medical Gazette*, 21 May 1900, 185.

76 Dr Stribal to Imperial and Royal Maritime Authorities, Trieste, 30 November 1899, British National Archives (TNA): MH 19/257.

77 A. John Gregory to the Under Colonial Secretary, 7 March 1900, TNA: MH 19/257.

78 Dr Doriga, ‘The Prevention of Plague Through the Suppression of Rats and Mice’, *Public Health*, January 1900, 291.

79 Walter Wyman, *The Bubonic Plague* (Washington: United States Government Printing Office, 1900), 46.

80 Burkhard Schnepel, ‘The Making of a Hub Society: Mauritius’ Path from Port of Call to Cyber Island’, in *Connectivity in Motion: Island Hubs in the Indian Ocean World*, eds. Burkhard Schnepel and Edward A. Alpers (Cham: Palgrave Macmillan, 2018), 236.

81 Palmer, Bradshaw, and Clark, ‘The Mauritius Register: Historical, Official & Commercial, Corected to the 30th June 1859’, lxiii.

82 Robert White Stevens, *On the Stowage of Ships and Their Cargoes, Freights, Charter-Parties, &c* (London: Longmans, 1859), 281.

83 Thomas Wilson, ‘Protector for Vessels Against Rats’, 24 January 1888. National Archives and Records Administration, United States (NARA) RG90 Central File, 1897–-1923, 544 Box 066.

84 Thos E. Heenan, ‘Russia. Device to Prevent Rats Coming Ashore from Ships’, *Public Health Reports (1896–1970)* 14, no. 27 (7 July 1899): 1820.

85 ’Ship-Borne Rats and Plague’, *The Lancet*, 4 May 1901, 1294. See also ‘Ship-Borne Rats and Plague’, W.H. Power, April 1901, TNA: MH 19/274; and Walter J. Howell to Secretary, Local Government Board, Whitehall, 19 October 1901, TNA: MH/274.

86 Noel Janisch to The Colonial Secretary’s Office, 18 February 1901, Western Cape Archives and Records Service (WCARS), CHB 234.

87 ’Ship-berthing Regulations which had been in operation at Melbourne and Sydney and which were adopted at the Inter-Colonial Plague Conference held at Melbourne in April, 1900’, TNA, MH 19/274.

88 ’A General Meeting of the Queensland Branch of the British Medical Association Was Held on March 2nd, 1900’, *The Australasian Medical Gazette*, 20 March 1900, 123–24.

89 See for example Major J. Crimmin’s testimony, which claims rats died on board one ship due to carbolic acid poisoning rather than plague (34–38). *Minutes of Evidence Taken by the Indian Plague Commission*, vol. 1 (London: Her Majesty’s Stationary Office, 1899); *Report of the Indian Plague Commission with Appendices and Summary*, vol. 5 (London: Her Majesty’s Stationary Office, 1901), 116–18. In the commissions’ summary of findings, likewise, the role played by rats is downplayed.

90 See F.M. Gibson (37), P.R. Cadell (143), Major R.H. Charles (252), *Minutes of Evidence Taken by the Indian Plague Commission*, 1899; J. Nield Cok, ‘Appendix No XX, Report on Plague in Calcutta’, in *Minutes of Evidence Taken by the Indian Plague Commission* (London: Her Majesty’s Stationary Office, 1899), 464; see also Captain G.S. Thompson (180) in *Minutes of Evidence Taken by the Indian Plague Commission*, vol. 3 (London: Her Majesty’s Stationary Office, 1900), 180.

91 *Minutes of Evidence Taken by the Indian Plague Commission*, 1900, 3:88.

92 Charles L. Bevans, ‘International Sanitary Convention’, in *Treaties and Other International Agreements of the United States of America 1776–1949* (Washington: United States Department of State, 1968), 359.

93 In addition, signatories were advised to destroy rats on vessels where infection was suspected, but not yet confirmed. Theodore Thomson, trans., *The International Sanitary Convention of Paris, 1903* (London: His Majesty’s Stationary Office, 1903), 6–8.

94 Ibid., 8.

95 Engelmann and Lynteris, *Sulphuric Utopias*, 142.

96 ’A sanitary convention signed ad referendum on Oct. 14, 1905, by the delegates of the United States, Chile, Coasta [Sic] Rica, Cuba, the Dominican Republic, Ecuador, Guatemala, Mexico, Nicaragua, Peru and Venesuela [sic] in New York Public Library, Elihu Root Collection of United States Documents. Accessible: https://www.google.co.uk/books/edition/Elihu_Root_Collection_of_United_States_D/2ntQAAAAYAAJ?hl=en&gbpv=0

97 In a circular from the United States Surgeon General to all ports in which US ships traded, various ports in China, Japan, South Africa and Australia all reported on the use of ratguards, while ports in Turkey, Austria, and the Congo either did not use them, choosing other strategies such as fumigation, or used them in select and minimal circumstances only. See NARA RG90 Central File 1897–1923, 537–544, Box 065.

98 Letter from the Health Officer of the Port of Bombay (J. Sladen), No. 1650, 13 July 1905, British Library (BL), India Office Records (IOR), IOR.P.7192.

99 Letter from the Political Resident, Aden, No. 7675, 16 December 1905, 1906. BL: IOR.P.7459.

100 G.D. Chitre, ‘A Report Summarizing Observations Carried Out at the Bombay Port Trust Docks by the Officer on Plague Prevention Inquiry, Under the Directions of the Director, Bombay Bacteriological Laboratory, Parel, Bombay’, p 3. ‘Fumigation at Indian Ports of Vessels Trading With South Afirca [sic] Dr. Chitres [sic Chritre] on Measure to Prevent the Access of Rates to Vessels With a View to Check the Outbreak of Players’ [sic], National Archives of India (NAI), Digitised by Abhilekh-Patal (A-P), https://www.abhilekh-patal.in/PR_000003006390, EDUCATION AND HEALTH_SANITARY_B_1923_MAR_61-65, Repository II, 1923–03.

101 Chitre, ‘A Report Summarizing Observations’, p 4.

102 ’Debates of the House of Representatives of Australia: Third Parliament, Second Session’ (Historic Hansard, 6 August 1907), https://historichansard.net/hofreps/1907/19070806_reps_3_37/#debate-9-s16.

103 ’Debates of the House of Representatives of Australia: Third Parliament, Second Session’ (Historic Hansard, 8 August 1907), https://historichansard.net/hofreps/1907/19070808_reps_3_37/#debate-13-s7.

104 Port Surgeon, Aden to First Assistant Resident, Aden, 19 October 1906, BL: IOR/R/20/A/2596.

105 Assistant Resident and Superintendent of Police, Aden to the First Assistant Resident of Aden, 2 March 1906; J.T. Galeha [illegible] to Inspector Vatcha, 23 February 1906, BL: IOR/R/20/A/2596.

106 Incidentally, under the Indian Ports Act of 1908, the use of “concave-convex” funnel-style guards was mandated in India, however “any other pattern of rat-guard” could also be approved by the government. *Report of the Bombay Chamber of Commerce for the Year 1915* (Bombay: The Times Press, 1915), 392.

107 This possibility was widely known. See for example ‘Ship Rats and the Spread of Plague’, *The Lancet*, 23 November 1912, 1448. A John Gregory to US Consul General in South Africa, 28 January 1909. NARA, RG90 Central File, 1897–1923, 537–544 Box 065.

108 G.D. Chitre, ‘A Report Summarizing Observations’, p 4. ‘Fumigation at Indian Ports of Vessels Trading With South Afirca’, NAI, A-P, PR_000003006390, EDUCATION AND HEALTH_SANITARY_B_1923_MAR_61-65, Repository II, 1923–-03.

109 Ibid., p 3.

110 Victor G. Heiser, *Annual Report of the Bureau of Health for the Philippine Islands for the Fiscal Year Ended June 30, 1912* (Manila, 1913), 27. In 1921, Chitre recommended that a similar but even larger design be used in Bombay, but the Port Health Officer regarded such a suggestion as impracticable in certain cases. Such guards were large and heavy, and could not accommodate ships that required multiple parallel ropes. Letter from the Port Health Officer of the Port of Bombay, no 2620, dated 21 December 1921, NAI, A-P, PR_000003006390.

111 F.E. Maynard to WC Rucker, 28 Aug 1912, NARA RG90 Central File 1897–1923, 544 Box 066.

112 Frederick E. Maynard [of Berkeley, California], 6 May 1913, ‘Trap-Guard’, *United States Patent Office*, NARA RG90 Central File 1897–1923, 544 Box 066.

113 W.C. Rucker to F.E. Maynard, 28 Aug 1912, NARA RG90 Central File, 1897–1923, 544 Box 066.

114 A design that resolved the short-circuiting problem was only invented in 1931. C.L. Bilderbeck, W.O.A. Young, and F.H. Otto, ‘Rat-Guards for Ships’ Hawsers’, *The Indian Medical Gazette*, January 1931, 18.

115 I have only been able to find occasional references to these guards. By contrast, extensive documentation exists for the use of circular and funnel guards.

116 For poisoning in ports, see Lucinda Cole, ‘Poison, Plagues, Dead Rats: A Multispecies History’, in *The Palgrave Handbook of Animals and Literature*, eds. Susan McHugh, Robert McKay, and John Miller (London: Palgrave Macmillan, 2021), 589–603.

117 Engelmann and Lynteris, *Sulphuric Utopias*, 176, 183–84.

118 ’Proceedings of the Department of Education and Health, May 1922: Prevention of yellow fever and exchange of health bulletins’, No. 352, 11 March 1921, p 28. NAI, A-P, Measures Against the Importation of the Yellow Fever in India Exchange of Health Bulletins, PR_000003005262, EDUCATION AND HEALTH_SANITARY_A_1922_MAY_25-43, Repository II.

119 ’Proceedings of the Department of Education and Health, May 1922: Prevention of Yellow Fever and exchange of Health Bulletins’, Pro. No. 39, NAI, A-P, Measures Against the Importation of the Yellow Fever in India Exchange of Health Bulletins, PR_000003005262, EDUCATION AND HEALTH_SANITARY_A_1922_MAY_25-43, Repository II.

120 Engelmann and Lynteris, *Sulphuric Utopias*, Chapter 7.

121 Guenter B. Risse, *Plague, Fear, and Politics in San Francisco’s Chinatown* (Baltimore: The Johns Hopkins University Press, 2012), 256–60; Joanna Leslie Dyl, *Seismic City: An Environmental History of San Francisco’s 1906 Earthquake*, Weyerhaeuser Environmental Books (Seattle London: University of Washington Press, 2017), Chapter 6.

122 Friench Simpson, ‘Methods of Plague Control’, *American Journal of Public Health*, 15 September 1920, 845–50; James Silver, *Farmers*’ *Bulletin No. 1533: Rat Control* (Washington: U.S. Department of Agriculture, 1927); Rupert Blue, ‘Bubonic Plague Control in California in 1903: Origin of Ratproofing as a Control Measure’, *California and Western Medicine* 40, no. 5 (May 1934): 363–65.

123 William Colby Rucker, ‘Rodent Extermination’, in *The Rat and Its Relation to Public Health*, ed. Public Health and Marine-Hospital Service of the United States (Washington: Government Printing Office, 1910), 161.

124 See for examples: Richard H. Creel, ‘Rat Proofing as an Antiplague Measure’, in *The Rat and Its Relation to Public Health*, ed. Public Health and Marine-Hospital Service of the United States (Washington: Government Printing Office, 1910), 171–78; Dawn Biehler, *Pests in the City: Flies, Bedbugs, Cockroaches, and Rats*, 2015.

125 S.B. Grubbs and B.E. Holsendorf, ‘The Rat-Proofing of Vessels’, *Public Health Reports* 40, no. 29 (1925): 1507.

126 Grubbs and Holsendorf, 1507.

127 Ibid.

128 ’RAT-PROOFED LINERS ARE CALLED A SUCCESS’, *New York Times*, 29 May 1927.

129 WT Butterwick to All Shipbuilders, Ministry of War Transport, 14 July 1944, TNA: MT 9/4951; ‘Ratproofing’ [Undated. Likely 1948]; TNA: MT 9/4653.

130 Grubbs and Holsendorf, ‘The Rat-Proofing of Vessels’, 1508.

131 Ibid.

132 On nineteenth-century ratcatchers, interspecies senses, and spatiality see: Neil Pemberton, ‘The Rat-Catcher’s Prank: Interspecies Cunningness and Scavenging in Henry Mayhew’s London’, *Journal of Victorian Culture* 19, no. 4 (2 October 2014): 520–35. For targeted fumigation of spaces rodents tended to infest, see Engelmann and Lynteris, *Sulphuric Utopias*, 196–97.

133 Grubbs and Holsendorf, ‘The Rat-Proofing of Vessels’, 1509.

134 Ibid.

135 Grubbs and Holsendorf, ‘The Rat-Proofing of Vessels’, 1510–14. Quote on 1509.

136 C.L. Williams, ‘Rat Infestation Inspection of Vessels’, *Public Health Reports* 47, no. 14 (1 April 1932): 769.

137 See NARA RG90, Box 7–8, ‘Ratproofing’.

138 According to evidence from New York and New Orleans. S.B. Grubbs, ‘Bubonic Plague and Maritime Quarantine: A Suggested System of Plague Control, Assuming That There Is Infectible and Noninfectible Territory, Discussing the Cheopis Index as a Measure of Infectibility, and Advocating the Rat-Proofing of Ships to Prevent the Spread of Plague by Sea’, *Public Health Reports* 42, no. 32 (1927): 2051.

139 Ibid., 2052.

140 Grubbs, 2052.

141 C.L. Williams, ‘A Rat and a Rat-Flea Survey of Ships at the Port of New York’, *Public Health Reports* 44, no. 9 (1929): 469.

142 J. Alexander Mitchell, ‘Tunnel Rat-Trap for Stores and Ships’, *Annals of Tropical Medicine & Parasitology* 23, no. 4 (31 December 1929): 446.

143 Ibid.

144 J. Alexander Mitchell, ‘Un Piege-Tunnel A Rats Pour Magasins et Navires’, *Office International D’Hygiène Publique* 21, no. 8 (1929): 1380–86.

145 Mitchell, ‘Tunnel Rat-Trap for Stores and Ships’, 447.

146 Vernon B. Link, ‘Plague on the High Seas’, *Public Health Reports (1896–-1970)* 66, no. 5 (9 November 1951): 1470.

147 S.B. Grubbs and B.E. Holsendorf, *The Rat Proofing of Vessels*, 3rd ed. (Washington: United States Government Printing Office, 1931), v.

148 Ibid., 1.

149 Engelmann and Lynteris, *Sulphuric Utopias*.

150 ’Deratisation Certificate’ and ‘Instructions to Port Medical Officers of Health in regard to the issue of deratisation certificate and deratisation exemption certificate’. *Office International D’Hygiene Publique*, 29 February 1928, BL: IOR, R/20/A/2592.

151 ’American Marine Standards Committee, “Rat Proofing of Ships: General Instructions”, Approved February 8, 1929’, in *American Marine Standards* (Washington: Government Printing Office, 1931), 1–5.

152 Samuel B. Grubbs, ‘Pan-Pacific Science Meeting, Honolulu, Friday, April 7, 1933’, *Journal of the Pan-Pacific Research Institution* 8, no. 3 (1933): 12.

153 *Annual Report of the Surgeon General of the Public Health Service of the United States for the Fiscal Year 1931* (Washington: Government Printing Office, 1931), 136–37.

154 ’Office International D’Hygiene Publique. Session of May, 1937. Report of the Quarantine Commission.’ TNA: MH 19/261.

155 ’Overseas Transmission of Bubonic Plague: A Danger Almost Eliminated’, *Public Health Reports* 52, no. 14 (1937): 412.

156 Ibid., 413.

157 G.L. Dunnahoo, extract from a letter, appended to T. Findlay to Secretary of the Ministry of Transport, 5 Feb 1948. TNA: MT 9/4951.

158 Knud Stowman, ‘International Sanitary Regulations’, *Public Health Reports (1896–1970)* 67, no. 10 (October 1952): 975.

159 C.V. Akin, ‘Comments on a Memorandum from Mr. G.M. Paterson, Naval Architect to the Chairman of the Shipbuilding Committee’, Merseyside Maritime Museum Archives, B/CUN/3/6/3/19.

